# 5,15-Bis(3,5-di-*tert*-butyl­phen­yl)-10,20-bis­(phenyl­ethyn­yl)porphyrin

**DOI:** 10.1107/S1600536809045954

**Published:** 2009-11-07

**Authors:** Hee Jung Kim, Atul P. Singh, Hee-Joon Kim

**Affiliations:** a Department of Applied Chemistry, Kumoh National Institute of Technology, 1 Yangho-dong, Gumi 730-701, Republic of Korea

## Abstract

In the centrosymmetric title compound, C_64_H_62_N_4_, the two phenyl­ethynyl groups lie at diagonal *meso* positions. The 24-membered porphyrin has in-plane distortion with respect to the mean plane of the macrocycle and two intra-ring bifurcated N—H⋯(N,N) hydrogen bonds occur. The dihedral angles between the phenyl rings in the phenyl­ethynyl group and the 3,5-bis­(*tert*-but­yl)phenyl group with respect to the mean plane of the porphyrin are 17.2 (2) and 59.2 (3)°. The *tert*-butyl groups are disordered over two sets of sites in a 0.661 (13):0.339 (13) ratio.

## Related literature

For background to porphyrin structures and electronic properties, see: Anderson *et al.* (1994 ▶, 1998 ▶); Fujita *et al.* (1995 ▶); Henari *et al.* (1997 ▶); Huuskonen *et al.* (1998 ▶); LeCours *et al.* (1996 ▶); Screen *et al.* (2002 ▶); Seo *et al.* (2008 ▶); Silvers & Tulinsky (1967 ▶).
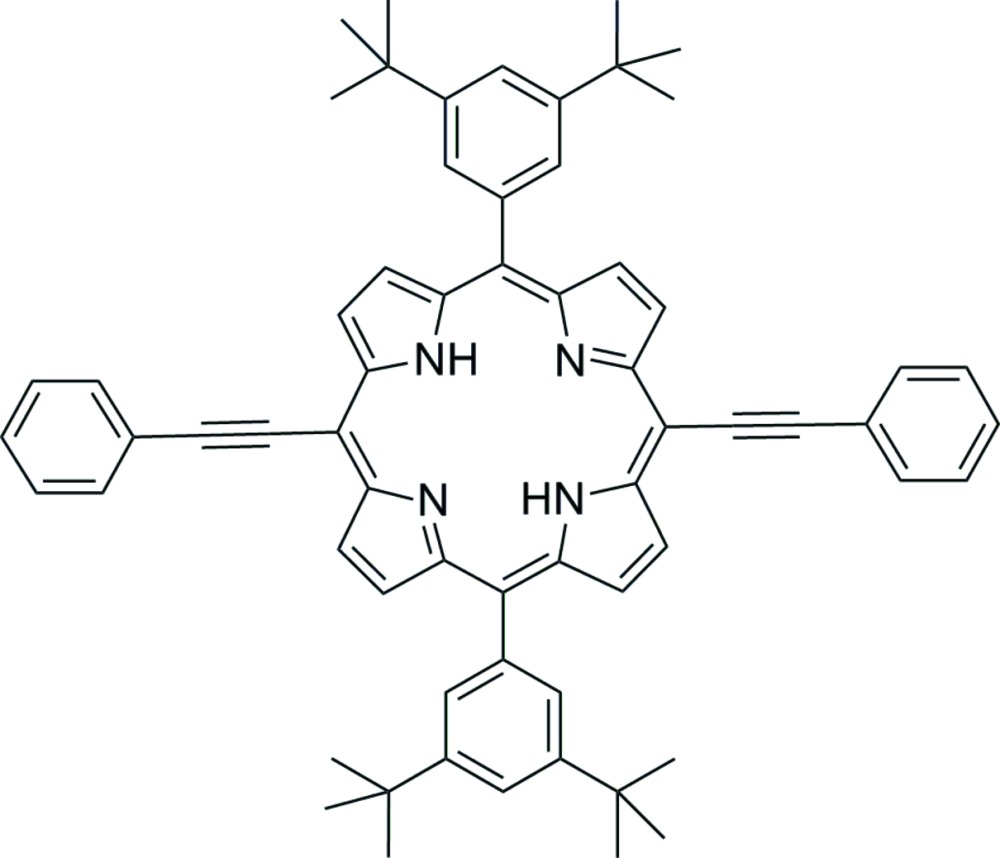


## Experimental

### 

#### Crystal data

C_64_H_62_N_4_
*M* *_r_* = 887.18
Triclinic, 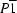

*a* = 9.9598 (19) Å
*b* = 10.496 (2) Å
*c* = 13.925 (3) Å
α = 86.236 (4)°
β = 80.266 (4)°
γ = 82.765 (4)°
*V* = 1421.8 (5) Å^3^
*Z* = 1
Mo *K*α radiation
μ = 0.06 mm^−1^
*T* = 213 K
0.45 × 0.10 × 0.05 mm

#### Data collection

Bruker SMART CCD diffractometer
Absorption correction: multi-scan (*SADABS*; Bruker, 2003 ▶) *T*_min_ = 0.974, *T*_max_ = 0.997
6144 measured reflections
4136 independent reflections
2068 reflections with *I* > 2σ(*I*)
*R*_int_ = 0.101
θ_max_ = 23.5°

#### Refinement

*R*[*F* ^2^ > 2σ(*F* ^2^)] = 0.107
*wR*(*F* ^2^) = 0.315
*S* = 0.99
4136 reflections
363 parameters
193 restraints
H-atom parameters constrained
Δρ_max_ = 1.13 e Å^−3^
Δρ_min_ = −0.30 e Å^−3^

### 

Data collection: *SMART* (Bruker, 2003 ▶); cell refinement: *SAINT* (Bruker, 2003 ▶); data reduction: *SAINT*; program(s) used to solve structure: *SHELXS97* (Sheldrick, 2008 ▶); program(s) used to refine structure: *SHELXL97* (Sheldrick, 2008 ▶); molecular graphics: *SHELXTL* (Sheldrick, 2008 ▶); software used to prepare material for publication: *SHELXTL* and *PLATON* (Spek, 2009 ▶).

## Supplementary Material

Crystal structure: contains datablocks I, global. DOI: 10.1107/S1600536809045954/hb5134sup1.cif

Structure factors: contains datablocks I. DOI: 10.1107/S1600536809045954/hb5134Isup2.hkl

Additional supplementary materials:  crystallographic information; 3D view; checkCIF report

## Figures and Tables

**Table 1:** Hydrogen-bond geometry (Å, °)

*D*—H⋯*A*	*D*—H	H⋯*A*	*D*⋯*A*	*D*—H⋯*A*
N1—H1*A*⋯N2	0.87	2.44	2.972 (6)	120
N1—H1*A*⋯N2^i^	0.87	2.35	2.891 (5)	121

Symmetry code: (i) 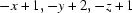.

**Fig. 1.**
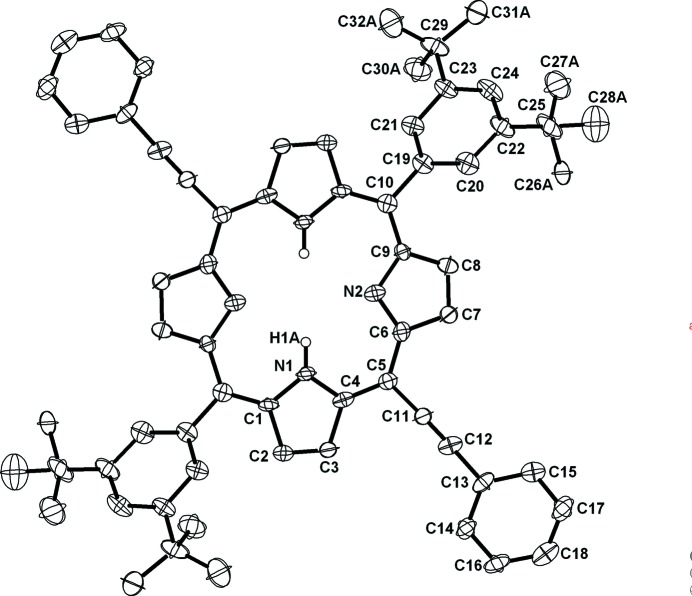
The molecular structure of (I) showing 30% probability displacement ellipsoids. Hydrogen atoms (except to N—H) have been omitted for the clarity.

**Fig. 2.**
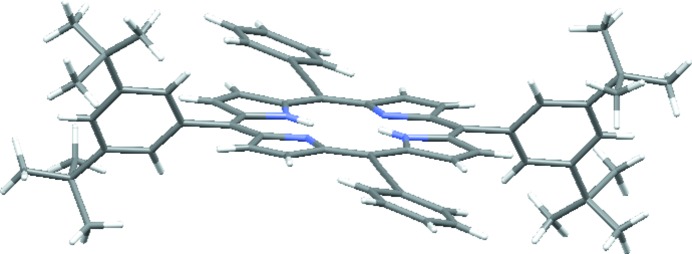
A view of molecular structure of (I) showing the tilted planes of the different aryl groups.

## supplementary crystallographic information

### Comment

The electronic and steric tunnings through surrogating various substituents on the *meso*- and beta-carbons play crucial role in the synthesis of diverse porphyrin systems. In this respect, *meso*-ethynyl porphyrins have attracted the attention due to their wide utilization for the development of conjugated electronic (LeCours *et al.*, 1996; Anderson *et al.*, 1994; Henari *et al.*, 1997; Screen *et al.*, 2002) and light harvesting materials. As an extension of our research on the conjugated photoelectronic materials (Seo *et al.*, 2008), the title compound (I) was prepared and its crystal structure determined.

The molecular structure of C64H62N4 (I) is shown in Figure 1. The structure shows symmetric molecular system due to presence of inversion center (Ci) in the core of porphyrin macrocycle. On assuming all four nitrogen atoms of all pyrolic groups in a plane, it was observed that the *meso*-carbons were deviated with ± 0.072 Å from the least-squares plane and find analogy with earlier reported compound 5,15-bis(3,5-di-*tert*-butylphenyl) -10,20-bis(trimethylsilylethynyl)porphyrin (Huuskonen *et al.*, 1998). The structural analysis of the porphyrin macrocycle reveals that the plane of phenyl rings in the phenylethynyl groups is slightly twisted with the dihedral angle of 17.17° with respect to the least-squares plane of the porphyrin, in contrast the plane of aryl rings associated with 3,5-bis(*tert*-butyl)phenyl groups (59.19°) (Figure 2). The dihedral angle associated with phenylethynyl groups (17.17°) is also much smaller with respect to phenyl planes slanting (61–63°) in tetraphenylporphyrin (Silvers *et al.*, 1967). The comparison of this result with the tetraphenylporphyrin, it was observed that on increasing the conjugation with phenyl groups leads to release of steric strain. The distances between the nitrogen atoms (N1—N2 = 2.972, N1—N2' = 2.891, N1—N1' = 4.167, N2—N2' = 4.126 Å) involve in the formation of basal parallelogram based core along with the C—C (triple bond) (1.165 Å) bond lengths and C(*meso*)-C(alpha)-C(beta) angles (178.07°) involving ethynyl groups show the analogy with previous reports (Fujita *et al.*, 1995; Huuskonen *et al.*, 1998). In addition, the distances involving the diagonal *meso*-carbon atoms (C5—C5' = 6.815, C10—C10' = 6.994 Å) also differ to each other and correlate with the previous work (Huuskonen *et al.*, 1998). The shortest intermolecular distances for the pi-pi and pi-H interactions are not observed less than 6.085 and 3.84 Å, respectively and imply the steric strain between aryl groups of adjacent porphyrin prevent the strong interactions in between adjacent molecular system (Anderson *et al.*, 1998).

### Experimental

The title compound was prepared from the corresponding dipyrromethane and phenylpropargyl aldehyde as follows. BF3.OEt2 (25 ML, 0.2 mmol) was added to a solution of *meso*-(3,5-di-*tert*-butylphenyl) dipyrromethane (0.669 g, 2.0 mmol) and phenylpropargyl aldehyde (245 ML, 2.0 mmol) in dry CH2Cl2 (200 ml). The reaction mixture was stirred for 10 min at room temperature.

2,3-Dichloro-5,6-dicyano-*p*-benzoquinone (340 mg, 0.15 mmol) was added and further stirred for 30 min. After evaporation of solvent to dryness, the title compound was separated by column chromatography (SiO2, CH2Cl2:n-hexane = 1:4). Recrystallization from a CH2Cl2/CH3CN solution afforded purple crystalline solid. Yield: 124 mg (14%). 1H NMR (200 MHz, CDCl3): Δ 9.70 (d, 4H), 8.89 (d, 4H), 8.06 (s, 4H), 8.00 (t, 2H), 7.82 (s, 2H), 1.55 (s, 36H), -1.92 (br, 2H). UV-vis (CH2Cl2): Λmax (log E) 443 (6.04), 554 (4.38), 594(4.92), 623 (4.25), 681 (4.61) nm.

Purple needles of (I) were grown by slow diffusion of CH_3_CN to a CH_2_Cl_2_ solution of the title compound.

### Refinement

The carbon atoms C26—C28 and C30—C32 and their attached H atoms are disordered over two sets of sites in a 66.1:33.1 ratio with total site occupancy of 1.00 for each one of them. The contributions of the mostly disordered solvent molecules were removed from the diffraction data using the SQUEEZE routine of *PLATON* software (Spek, 2009), and then final refinements were carried out. All the non-hydrogen atoms were refined anisotropically, and hydrogen atoms were placed in their geometrically ideal positions.

### Crystal data

**Table:**

C_64_H_62_N_4_	*Z* = 1
*M**_r_* = 887.18	*F*(000) = 474
Triclinic, *P*1	*D*_x_ = 1.036 Mg m^−^^3^
Hall symbol: -P 1	Mo *K*α radiation, λ = 0.71073 Å
*a* = 9.9598 (19) Å	Cell parameters from 4136 reflections
*b* = 10.496 (2) Å	θ = 2.0–23.5°
*c* = 13.925 (3) Å	µ = 0.06 mm^−^^1^
α = 86.236 (4)°	*T* = 213 K
β = 80.266 (4)°	Needle, purple
γ = 82.765 (4)°	0.45 × 0.10 × 0.05 mm
*V* = 1421.8 (5) Å^3^	

### Data collection

**Table:**

Bruker SMART CCD diffractometer	4136 independent reflections
Radiation source: fine-focus sealed tube	2068 reflections with *I* > 2σ(*I*)
graphite	*R*_int_ = 0.101
ω scans	θ_max_ = 23.5°, θ_min_ = 2.0°
Absorption correction: multi-scan (*SADABS*; Bruker, 2003)	*h* = −8→11
*T*_min_ = 0.974, *T*_max_ = 0.997	*k* = −11→11
6144 measured reflections	*l* = −11→15

### Refinement

**Table:**

Refinement on *F*^2^	Primary atom site location: structure-invariant direct methods
Least-squares matrix: full	Secondary atom site location: difference Fourier map
*R*[*F*^2^ > 2σ(*F*^2^)] = 0.107	Hydrogen site location: inferred from neighbouring sites
*wR*(*F*^2^) = 0.315	H-atom parameters constrained
*S* = 0.99	*w* = 1/[σ^2^(*F*_o_^2^) + (0.1906*P*)^2^] where *P* = (*F*_o_^2^ + 2*F*_c_^2^)/3
4136 reflections	(Δ/σ)_max_ < 0.001
363 parameters	Δρ_max_ = 1.13 e Å^−^^3^
193 restraints	Δρ_min_ = −0.30 e Å^−^^3^

### Fractional atomic coordinates and isotropic or equivalent isotropic displacement parameters (Å^2^)

**Table:**

	*x*	*y*	*z*	*U*_iso_*/*U*_eq_	Occ. (<1)
N1	0.4997 (4)	0.8413 (3)	0.5981 (3)	0.0445 (11)	
H1A	0.4940	0.9116	0.5616	0.053*	
N2	0.3805 (4)	0.9196 (3)	0.4184 (3)	0.0481 (11)	
C1	0.5695 (5)	0.8204 (4)	0.6753 (3)	0.0429 (13)	
C2	0.5537 (6)	0.6929 (5)	0.7151 (4)	0.0529 (14)	
H2	0.5907	0.6530	0.7689	0.063*	
C3	0.4771 (6)	0.6413 (5)	0.6622 (4)	0.0600 (16)	
H3	0.4511	0.5578	0.6718	0.072*	
C4	0.4409 (5)	0.7342 (4)	0.5880 (4)	0.0449 (13)	
C5	0.3646 (5)	0.7155 (4)	0.5160 (4)	0.0482 (13)	
C6	0.3377 (5)	0.8019 (5)	0.4380 (4)	0.0468 (13)	
C7	0.2576 (6)	0.7764 (5)	0.3646 (4)	0.0577 (15)	
H7	0.2154	0.7020	0.3608	0.069*	
C8	0.2563 (6)	0.8818 (5)	0.3030 (4)	0.0564 (15)	
H8	0.2137	0.8929	0.2472	0.068*	
C9	0.3299 (5)	0.9725 (4)	0.3367 (3)	0.0409 (12)	
C10	0.3527 (5)	1.0946 (5)	0.2919 (3)	0.0470 (13)	
C11	0.3101 (6)	0.5928 (5)	0.5201 (4)	0.0557 (14)	
C12	0.2674 (6)	0.4937 (5)	0.5260 (4)	0.0606 (16)	
C13	0.2183 (6)	0.3674 (5)	0.5331 (4)	0.0584 (15)	
C14	0.2721 (6)	0.2699 (5)	0.5915 (4)	0.0605 (15)	
H14	0.3410	0.2849	0.6265	0.073*	
C15	0.1152 (6)	0.3433 (5)	0.4811 (4)	0.0626 (16)	
H15	0.0781	0.4072	0.4393	0.075*	
C16	0.2265 (7)	0.1512 (5)	0.5990 (5)	0.0712 (18)	
H16	0.2655	0.0850	0.6381	0.085*	
C17	0.0704 (7)	0.2243 (6)	0.4930 (4)	0.0760 (19)	
H17	−0.0007	0.2083	0.4605	0.091*	
C18	0.1249 (8)	0.1287 (6)	0.5501 (5)	0.0757 (19)	
H18	0.0929	0.0475	0.5558	0.091*	
C19	0.2853 (6)	1.1365 (5)	0.2057 (4)	0.0511 (14)	
C20	0.1439 (6)	1.1470 (5)	0.2137 (4)	0.0636 (16)	
H20	0.0914	1.1283	0.2745	0.076*	
C21	0.3627 (6)	1.1657 (4)	0.1160 (4)	0.0541 (14)	
H21	0.4586	1.1596	0.1104	0.065*	
C22	0.0761 (6)	1.1849 (6)	0.1337 (4)	0.0704 (17)	
C23	0.3015 (6)	1.2037 (5)	0.0341 (4)	0.0612 (15)	
C24	0.1597 (6)	1.2114 (6)	0.0466 (4)	0.0714 (18)	
H24	0.1172	1.2366	−0.0081	0.086*	
C25	−0.0782 (7)	1.2010 (8)	0.1463 (5)	0.090 (2)	
C26A	−0.1491 (10)	1.1396 (11)	0.2354 (9)	0.077 (3)	0.661 (13)
H26A	−0.1029	1.0541	0.2459	0.115*	0.661 (13)
H26B	−0.2433	1.1337	0.2283	0.115*	0.661 (13)
H26C	−0.1479	1.1907	0.2908	0.115*	0.661 (13)
C27A	−0.1397 (14)	1.3240 (12)	0.1232 (13)	0.118 (5)	0.661 (13)
H27A	−0.2385	1.3240	0.1324	0.178*	0.661 (13)
H27B	−0.1063	1.3481	0.0558	0.178*	0.661 (13)
H27C	−0.1171	1.3852	0.1655	0.178*	0.661 (13)
C28A	−0.1218 (15)	1.1113 (16)	0.0638 (12)	0.147 (6)	0.661 (13)
H28A	−0.0845	1.0225	0.0739	0.221*	0.661 (13)
H28B	−0.0852	1.1424	−0.0013	0.221*	0.661 (13)
H28C	−0.2209	1.1172	0.0711	0.221*	0.661 (13)
C26B	−0.125 (4)	1.110 (3)	0.176 (3)	0.156 (15)	0.339 (13)
H26D	−0.2235	1.1246	0.1789	0.234*	0.339 (13)
H26E	−0.1041	1.0889	0.2416	0.234*	0.339 (13)
H26F	−0.0867	1.0400	0.1344	0.234*	0.339 (13)
C27B	−0.121 (3)	1.3362 (18)	0.2041 (19)	0.109 (9)	0.339 (13)
H27D	−0.0753	1.4043	0.1668	0.164*	0.339 (13)
H27E	−0.0933	1.3249	0.2679	0.164*	0.339 (13)
H27F	−0.2195	1.3590	0.2115	0.164*	0.339 (13)
C28B	−0.117 (3)	1.263 (3)	0.048 (2)	0.153 (12)	0.339 (13)
H28D	−0.0526	1.3227	0.0203	0.229*	0.339 (13)
H28E	−0.2091	1.3088	0.0598	0.229*	0.339 (13)
H28F	−0.1147	1.1962	0.0025	0.229*	0.339 (13)
C29	0.3836 (7)	1.2398 (6)	−0.0623 (4)	0.0763 (18)	
C30A	0.495 (6)	1.131 (5)	−0.084 (4)	0.094 (9)	0.121 (8)
H30A	0.5050	1.0805	−0.0243	0.141*	0.121 (8)
H30B	0.5801	1.1652	−0.1102	0.141*	0.121 (8)
H30C	0.4709	1.0776	−0.1311	0.141*	0.121 (8)
C31A	0.294 (5)	1.274 (6)	−0.138 (4)	0.089 (10)	0.121 (8)
H31A	0.3509	1.2927	−0.2001	0.134*	0.121 (8)
H31B	0.2299	1.3483	−0.1193	0.134*	0.121 (8)
H31C	0.2446	1.2018	−0.1446	0.134*	0.121 (8)
C32A	0.445 (7)	1.358 (5)	−0.035 (5)	0.110 (10)	0.121 (8)
H32A	0.5169	1.3299	0.0039	0.165*	0.121 (8)
H32B	0.3740	1.4150	0.0026	0.165*	0.121 (8)
H32C	0.4841	1.4041	−0.0938	0.165*	0.121 (8)
C30B	0.3606 (9)	1.1477 (8)	−0.1410 (5)	0.092 (3)	0.879 (8)
H30D	0.4134	1.1703	−0.2035	0.138*	0.879 (8)
H30E	0.2640	1.1563	−0.1463	0.138*	0.879 (8)
H30F	0.3903	1.0595	−0.1217	0.138*	0.879 (8)
C31B	0.3428 (10)	1.3783 (8)	−0.0936 (6)	0.109 (3)	0.879 (8)
H31D	0.3979	1.3990	−0.1557	0.163*	0.879 (8)
H31E	0.3579	1.4346	−0.0449	0.163*	0.879 (8)
H31F	0.2466	1.3899	−0.1003	0.163*	0.879 (8)
C32B	0.5416 (8)	1.2160 (9)	−0.0616 (5)	0.086 (2)	0.879 (8)
H32D	0.5902	1.2415	−0.1249	0.130*	0.879 (8)
H32E	0.5680	1.1255	−0.0475	0.130*	0.879 (8)
H32F	0.5645	1.2664	−0.0120	0.130*	0.879 (8)

### Atomic displacement parameters (Å^2^)

**Table:**

	*U*^11^	*U*^22^	*U*^33^	*U*^12^	*U*^13^	*U*^23^
N1	0.048 (3)	0.031 (2)	0.050 (3)	−0.0016 (18)	−0.004 (2)	0.0123 (18)
N2	0.053 (3)	0.039 (2)	0.050 (3)	−0.0027 (19)	−0.002 (2)	0.0033 (19)
C1	0.043 (3)	0.033 (3)	0.046 (3)	0.006 (2)	−0.001 (2)	0.012 (2)
C2	0.057 (4)	0.043 (3)	0.059 (3)	−0.008 (3)	−0.014 (3)	0.014 (3)
C3	0.058 (4)	0.047 (3)	0.071 (4)	−0.009 (3)	−0.006 (3)	0.026 (3)
C4	0.050 (3)	0.032 (3)	0.048 (3)	−0.005 (2)	0.005 (3)	0.006 (2)
C5	0.049 (3)	0.041 (3)	0.051 (3)	−0.007 (2)	−0.002 (3)	0.006 (2)
C6	0.035 (3)	0.050 (3)	0.051 (3)	−0.001 (2)	0.001 (2)	−0.002 (2)
C7	0.061 (4)	0.055 (3)	0.059 (4)	−0.021 (3)	−0.006 (3)	0.004 (3)
C8	0.061 (4)	0.066 (4)	0.043 (3)	−0.014 (3)	−0.006 (3)	0.000 (3)
C9	0.035 (3)	0.039 (3)	0.046 (3)	0.000 (2)	−0.004 (2)	0.001 (2)
C10	0.043 (3)	0.050 (3)	0.043 (3)	0.003 (2)	0.000 (2)	−0.001 (2)
C11	0.059 (4)	0.046 (3)	0.060 (4)	−0.014 (3)	−0.004 (3)	0.011 (3)
C12	0.071 (4)	0.046 (3)	0.062 (4)	−0.012 (3)	−0.001 (3)	0.008 (3)
C13	0.056 (4)	0.043 (3)	0.071 (4)	−0.009 (3)	0.007 (3)	−0.008 (3)
C14	0.055 (4)	0.049 (3)	0.075 (4)	−0.007 (3)	−0.004 (3)	−0.001 (3)
C15	0.071 (4)	0.054 (3)	0.059 (4)	−0.006 (3)	−0.004 (3)	0.000 (3)
C16	0.065 (4)	0.044 (3)	0.093 (5)	0.001 (3)	0.009 (4)	0.011 (3)
C17	0.090 (5)	0.084 (5)	0.058 (4)	−0.038 (4)	0.000 (3)	−0.014 (3)
C18	0.080 (5)	0.055 (4)	0.085 (5)	−0.020 (3)	0.016 (4)	−0.008 (4)
C19	0.053 (4)	0.052 (3)	0.046 (3)	0.003 (3)	−0.008 (3)	−0.005 (2)
C20	0.049 (4)	0.076 (4)	0.057 (4)	0.011 (3)	0.004 (3)	−0.001 (3)
C21	0.054 (4)	0.053 (3)	0.049 (3)	0.007 (3)	−0.002 (3)	−0.003 (2)
C22	0.057 (4)	0.086 (4)	0.061 (4)	0.020 (3)	−0.010 (3)	0.000 (3)
C23	0.062 (4)	0.062 (3)	0.057 (4)	0.011 (3)	−0.018 (3)	0.004 (3)
C24	0.063 (5)	0.095 (4)	0.050 (4)	0.024 (3)	−0.015 (3)	0.000 (3)
C25	0.054 (5)	0.121 (6)	0.086 (5)	0.029 (4)	−0.020 (4)	0.009 (4)
C26A	0.038 (5)	0.093 (6)	0.096 (7)	−0.002 (4)	−0.014 (5)	0.018 (5)
C27A	0.097 (8)	0.117 (8)	0.135 (9)	0.002 (6)	−0.020 (7)	0.011 (7)
C28A	0.109 (9)	0.171 (10)	0.167 (10)	−0.024 (7)	−0.031 (7)	−0.014 (8)
C26B	0.149 (17)	0.162 (17)	0.157 (17)	−0.013 (10)	−0.031 (10)	0.002 (10)
C27B	0.099 (11)	0.111 (11)	0.114 (12)	−0.006 (8)	−0.015 (9)	0.000 (9)
C28B	0.147 (15)	0.165 (15)	0.148 (14)	−0.006 (9)	−0.037 (9)	−0.006 (9)
C29	0.081 (4)	0.089 (4)	0.048 (3)	0.016 (3)	−0.002 (3)	0.004 (3)
C30A	0.089 (12)	0.096 (11)	0.092 (13)	0.003 (8)	−0.010 (9)	0.002 (9)
C31A	0.087 (12)	0.095 (14)	0.085 (12)	−0.007 (8)	−0.015 (9)	0.005 (9)
C32A	0.112 (15)	0.112 (12)	0.106 (15)	−0.011 (9)	−0.021 (10)	−0.012 (10)
C30B	0.083 (5)	0.133 (6)	0.052 (4)	0.007 (4)	0.000 (3)	−0.010 (4)
C31B	0.114 (6)	0.108 (5)	0.089 (5)	−0.002 (4)	0.002 (5)	0.039 (4)
C32B	0.083 (5)	0.122 (6)	0.045 (4)	0.001 (4)	0.003 (3)	0.009 (4)

### Geometric parameters (Å, °)

**Table:**

N1—C4	1.357 (6)	C29—C30A	1.49 (4)
N1—C1	1.367 (6)	C29—C31A	1.49 (4)
N2—C6	1.354 (6)	C29—C31B	1.515 (9)
N2—C9	1.378 (6)	C29—C30B	1.570 (10)
C1—C10^i^	1.397 (7)	C29—C32B	1.563 (10)
C1—C2	1.431 (6)	C29—C32A	1.55 (5)
C2—C3	1.329 (7)	N1—H1A	0.869 (3)
C3—C4	1.433 (7)	C2—H2	0.941 (6)
C4—C5	1.393 (7)	C3—H3	0.940 (5)
C5—C6	1.407 (7)	C8—H8	0.940 (6)
C5—C11	1.454 (7)	C14—H14	0.940 (6)
C6—C7	1.455 (7)	C15—H15	0.940 (5)
C7—C8	1.355 (7)	C16—H16	0.940 (6)
C8—C9	1.420 (7)	C17—H17	0.940 (7)
C9—C10	1.415 (6)	C18—H18	0.941 (7)
C10—C1^i^	1.397 (7)	C20—H20	0.940 (5)
C10—C19	1.488 (7)	C21—H21	0.939 (6)
C11—C12	1.164 (7)	C24—H24	0.940 (6)
C12—C13	1.462 (7)	C26A—H26A	0.97 (1)
C13—C14	1.377 (8)	C26A—H26B	0.97 (1)
C13—C15	1.409 (8)	C26A—H26C	0.97 (1)
C14—C16	1.371 (7)	C27A—H27A	0.97 (1)
C15—C17	1.369 (8)	C27A—H27B	0.97 (2)
C16—C18	1.364 (9)	C27A—H27C	0.97 (2)
C17—C18	1.355 (9)	C28A—H28A	0.97 (2)
C19—C20	1.385 (7)	C28A—H28B	0.97 (2)
C19—C21	1.390 (7)	C28A—H28C	0.97 (1)
C20—C22	1.408 (8)	C30A—H30A	0.97 (5)
C21—C23	1.395 (7)	C30A—H30B	0.97 (6)
C22—C24	1.383 (8)	C30A—H30C	0.97 (6)
C22—C25	1.506 (9)	C31A—H31A	0.97 (5)
C23—C24	1.386 (8)	C31A—H31B	0.96 (5)
C23—C29	1.502 (8)	C31A—H31C	0.97 (6)
C25—C26B	1.14 (3)	C32A—H32A	0.97 (7)
C25—C27A	1.400 (13)	C32A—H32B	0.97 (6)
C25—C26A	1.473 (12)	C32A—H32C	0.97 (6)
C25—C28B	1.57 (3)	N1—H1A	0.869 (3)
C25—C27B	1.651 (16)	C2—H2	0.941 (6)
C25—C28A	1.676 (16)		
			
C4—N1—C1	108.7 (4)	C28B—C25—C28A	59.0 (12)
C6—N2—C9	107.9 (4)	C27B—C25—C28A	143.9 (12)
N1—C1—C10^i^	126.7 (4)	C30A—C29—C31A	116 (3)
N1—C1—C2	107.9 (5)	C30A—C29—C23	106 (2)
C10^i^—C1—C2	125.4 (5)	C31A—C29—C23	112 (2)
C3—C2—C1	107.4 (5)	C30A—C29—C31B	142 (2)
C2—C3—C4	108.6 (5)	C31A—C29—C31B	58 (2)
N1—C4—C5	126.4 (4)	C23—C29—C31B	111.3 (5)
N1—C4—C3	107.4 (5)	C30A—C29—C30B	65 (2)
C5—C4—C3	126.1 (5)	C31A—C29—C30B	54 (2)
C4—C5—C6	126.8 (4)	C23—C29—C30B	109.0 (6)
C4—C5—C11	116.8 (4)	C31B—C29—C30B	110.0 (6)
C6—C5—C11	116.4 (5)	C30A—C29—C32B	44 (2)
N2—C6—C5	126.7 (5)	C31A—C29—C32B	136 (2)
N2—C6—C7	109.1 (4)	C23—C29—C32B	112.4 (5)
C5—C6—C7	124.2 (5)	C31B—C29—C32B	110.1 (7)
C8—C7—C6	105.9 (5)	C30B—C29—C32B	103.9 (6)
C7—C8—C9	108.7 (5)	C30A—C29—C32A	110 (3)
N2—C9—C8	108.4 (4)	C31A—C29—C32A	112 (3)
N2—C9—C10	125.5 (5)	C23—C29—C32A	101 (2)
C8—C9—C10	126.0 (5)	C31B—C29—C32A	54 (2)
C1^i^—C10—C9	124.6 (5)	C30B—C29—C32A	150 (2)
C1^i^—C10—C19	117.9 (4)	C32B—C29—C32A	66 (2)
C9—C10—C19	117.4 (5)	C25—C26A—H26A	110 (1)
C12—C11—C5	178.0 (6)	C25—C26A—H26B	109 (1)
C11—C12—C13	178.2 (6)	C25—C26A—H26C	109 (1)
C14—C13—C15	118.9 (5)	H26A—C26A—H26B	109 (1)
C14—C13—C12	120.2 (6)	H26A—C26A—H26C	109 (1)
C15—C13—C12	120.9 (5)	H26B—C26A—H26C	109 (1)
C16—C14—C13	120.7 (6)	C25—C27A—H27A	109 (1)
C17—C15—C13	118.2 (6)	C25—C27A—H27B	110 (1)
C18—C16—C14	120.3 (6)	C25—C27A—H27C	109 (1)
C18—C17—C15	122.4 (7)	H27A—C27A—H27B	109 (1)
C17—C18—C16	119.4 (6)	H27A—C27A—H27C	109 (1)
C20—C19—C21	118.9 (5)	H27B—C27A—H27C	109 (1)
C20—C19—C10	120.3 (5)	C25—C28A—H28A	109 (1)
C21—C19—C10	120.8 (5)	C25—C28A—H28B	109 (1)
C19—C20—C22	122.2 (5)	C25—C28A—H28C	109 (1)
C23—C21—C19	121.6 (5)	H28A—C28A—H28B	109 (2)
C24—C22—C20	115.8 (6)	H28A—C28A—H28C	110 (2)
C24—C22—C25	123.6 (6)	H28B—C28A—H28C	109 (2)
C20—C22—C25	120.6 (5)	C23—C29—C30A	106 (2)
C24—C23—C21	116.7 (5)	C23—C29—C31A	111 (2)
C24—C23—C29	121.1 (5)	C23—C29—C32A	101 (2)
C21—C23—C29	122.1 (6)	C30A—C29—C31A	116 (3)
C22—C24—C23	124.8 (6)	C30A—C29—C32A	110 (3)
C26B—C25—C27A	131 (2)	C31A—C29—C32A	112 (3)
C26B—C25—C26A	36.7 (19)	C29—C30A—H30A	109 (5)
C27A—C25—C26A	113.9 (9)	C29—C30A—H30B	109 (5)
C26B—C25—C22	113 (2)	C29—C30A—H30C	109 (5)
C27A—C25—C22	115.8 (9)	H30A—C30A—H30B	110 (5)
C26A—C25—C22	115.9 (6)	H30A—C30A—H30C	110 (5)
C26B—C25—C28B	117 (2)	H30B—C30A—H30C	110 (5)
C27A—C25—C28B	48.9 (12)	C29—C31A—H31A	109 (5)
C26A—C25—C28B	136.7 (14)	C29—C31A—H31B	110 (5)
C22—C25—C28B	106.5 (13)	C29—C31A—H31C	109 (5)
C26B—C25—C27B	120 (2)	H31A—C31A—H31B	110 (5)
C27A—C25—C27B	45.0 (9)	H31A—C31A—H31C	109 (5)
C26A—C25—C27B	85.5 (11)	H31B—C31A—H31C	110 (5)
C22—C25—C27B	102.9 (11)	C29—C32A—H32A	110 (5)
C28B—C25—C27B	93.6 (14)	C29—C32A—H32B	110 (5)
C26B—C25—C28A	64 (2)	C29—C32A—H32C	110 (5)
C27A—C25—C28A	102.6 (10)	H32A—C32A—H32B	109 (6)
C26A—C25—C28A	98.7 (9)	H32A—C32A—H32C	109 (6)
C22—C25—C28A	107.1 (7)	H32B—C32A—H32C	109 (6)

Symmetry codes: (i) −*x*+1, −*y*+2, −*z*+1.

### Hydrogen-bond geometry (Å, °)

**Table:**

*D*—H···*A*	*D*—H	H···*A*	*D*···*A*	*D*—H···*A*
N1—H1A···N2	0.87	2.44	2.972 (6)	120
N1—H1A···N2^i^	0.87	2.35	2.891 (5)	121

Symmetry codes: (i) −*x*+1, −*y*+2, −*z*+1.
